# A prospective study of mental health care for comorbid depressed mood in older adults with painful osteoarthritis

**DOI:** 10.1186/1471-244X-11-147

**Published:** 2011-09-12

**Authors:** Yehoshua Gleicher, Ruth Croxford, Jacqueline Hochman, Gillian Hawker

**Affiliations:** 1 Faculty of Medicine, University of Toronto, 1 Kings College Circle, Toronto, ON M5S 1A8, Canada; 2 Institute for Clinical Evaluative Sciences, G1 06, 2075 Bayview Avenue, Toronto, ON M4N 3M5, Canada; 3 Department of Health Policy, Management and Evaluation, University of Toronto, 155 College Street, Suite 425, Toronto, ON M5T 3M6, Canada; 4 Department of Medicine, Women's College Hospital, 76 Grenville Street, Toronto, ON M5S 1B2, Canada; 5 Women's College Research Institute, Women's College Hospital, 790 Bay Street, 7th Floor, Toronto, ON M5G 1N8, Canada

## Abstract

**Background:**

Comorbid depression is common among adults with painful osteoarthritis (OA). We evaluated the relationship between depressed mood and receipt of mental health (MH) care services.

**Methods:**

In a cohort with OA, annual interviews assessed comorbidity, arthritis severity, and MH (SF-36 mental health score). Surveys were linked to administrative health databases to identify mental health-related visits to physicians in the two years following the baseline interview (1996-98). Prescriptions for anti-depressants were ascertained for participants aged 65+ years (eligible for drug benefits). The relationship between MH scores and MH-related physician visits was assessed using zero-inflated negative binomial regression, adjusting for confounders. For those aged 65+ years, logistic regression examined the probability of receiving *any *MH-related care (physician visit or anti-depressant prescription).

**Results:**

Analyses were based on 2,005 (90.1%) individuals (mean age 70.8 years). Of 576 (28.7%) with probable depression (MH score < 60/100), 42.5% experienced one or more MH-related physician visits during follow-up. The likelihood of a physician visit was associated with sex (adjusted OR women vs. men = 5.87, p = 0.005) and MH score (adjusted OR per 10-point decrease in MH score = 1.63, p = 0.003). Among those aged 65+, 56.7% with probable depression received *any *MH care. The likelihood of receiving *any *MH care exhibited a significant interaction between MH score and self-reported health status (p = 0.0009); with good general health, worsening MH was associated with increased likelihood of MH care; as general health declined, this effect was attenuated.

**Conclusions:**

Among older adults with painful OA, more than one-quarter had depressed mood, but almost half received no mental health care, suggesting a care gap.

## Background

Osteoarthritis (OA) is a common, disabling, and costly disease[1-3]. Treatment has focused on ameliorating pain and reducing accompanying functional limitations[4]. Less attention has been given to the downstream effects of pain and disability on mood[5] - yet population and clinical studies consistently suggest that OA pain and disability are found together with depression more frequently than would be expected by chance[6-9]. Prospectively, we have shown that painful OA leads to depressed mood through the mediating effects of pain on fatigue and disability[10]. For those with painful OA, concomitant depression is associated with greater pain and disability[11], worse outcomes following knee replacement surgery[12], and greater health care use[13]. In other chronic pain conditions, comorbid depression has been linked to reduced adherence to pain interventions[14] and when used, reduced effectiveness of these therapies[15]. Thus, recognition and treatment of comorbid depression has the potential to improve outcomes for people with chronic painful OA [16]. Yet, mental health (MH) conditions are under-recognized and consequently, under-treated in older adults, the same population disproportionately affected by OA[17-19].

Despite the documented link between pain and depressed mood, few studies have examined the diagnosis and treatment of depressed mood in the setting of painful OA. The primary objective of this study was to evaluate, in a Canadian cohort with chronic symptomatic hip and knee OA, the relationship between depressed mood and mental health-related physician visits and anti-depressant prescription. *A priori*, we were interested in the proportion of participants who met criteria for probable depression who received any mental health-related care. We hypothesized that the prevalence of depressed mood would be high, but that the associated frequency of mental health-related physician visits would suggest under-recognition of concomitant depression.

## Methods

### Study Population

Participants were members of a longitudinal cohort of individuals with moderate-to-severe hip or knee OA. Details of cohort recruitment have been published previously[20]. Briefly, participants were recruited between 1996 and 1998 through a screening survey of 100% of the population 55+ years residing in two regions of Ontario, Canada, one rural and one urban. Individuals were selected for cohort inclusion if they: i) reported difficulty in the last three months with each of the following: stair climbing, rising from a chair, standing and walking; ii) swelling, pain or stiffness in any joint lasting at least six weeks; and iii) indicated on a diagram that a hip or knee had been 'troublesome'. Based on these criteria, a cohort of 2,411 individuals with arthritis was established. In a subsequent validation study, following re-administration of the screening questions, trained physiotherapists conducted a standardized examination of the hips and knees in 475 survey respondents (375 with and 100 without hip/knee complaints). Of the 372 validation study participants who met our screening criteria for hip/knee arthritis, 96% had clinical signs of hip and/or knee arthritis on examination[20].

### Assessments

Participation rates for the initial baseline surveys were 80.6% and 75.4% for the rural and urban regions, respectively. Follow-up, conducted annually by standardized telephone interviews, obtained information on sociodemographics (age, sex, race, level of education, annual household income, living circumstances), body mass index and severity of hip/knee symptoms and disability using the Western Ontario McMaster Universities OA Index (WOMAC) pain and function subscale and summary scores[21], for which higher scores indicate worse symptoms or disability. The SF-36, a self-administered multidimensional questionnaire, was used to assess health-related quality of life[22]. Participants indicated if they had seen a physician or taken any medication in the past year for each of 13 health problems. Prior treatment for depression or another mental health condition was also assessed. Ethical approval was obtained from Women's College Hospital Research Ethics Board and informed consent was acquired from all participants.

### Assessment of Depressed Mood

Mental health was assessed at baseline and then annually over the two-year study period using the mental health subscale of the SF-36 (MH score)[22]; higher scores indicate better mental health. Friedman et al. [23] have shown that MH scores < 60/100 are associated with clinical depression, as defined using the Mini-International Neuropsychiatric Interview-Major Depressive Episode module (MINI-MDE) (sensitivity = 78.7%, specificity = 72.1%). At one time-point during follow-up, data were obtained from cohort participants for both the MH score and the Center for Epidemiologic Studies Depression Scale (CES-D). The CES-D is a valid and reliable measure of depressed mood[24]; higher scores indicate more depressed mood. The Spearman correlation between SF-36 MH and CES-D scores was -0.77, p < 0.0001; further, a CES-D score ≥16, considered indicative of possible depression[24], corresponded to an SF-36 MH score ≤68. For the present study, a conservative cut-point score of 60 on the MH scale was used to categorize cohort participants as having depressed (scores < 60/100) or non-depressed mood (scores ≥60/100). Those meeting criteria for depressed mood were considered to have probable depression.

### Assessment of Mental Health-Related Health Service Utilization

Participants' survey data were linked to provincial administrative databases using unique anonymous patient identifiers[25]. In Ontario, visits to physicians are funded by the single-payer Ontario Health Insurance Plan (OHIP); further, the primary care physician (PCP) acts as the gatekeeper to specialized health care services, such that visits to mental health specialists are only accessible via referral from the PCP. For all cohort participants, we ascertained physician services (PCPs, and psychiatrists) using claims recorded in the OHIP Physician and Laboratory Billing Records. Each claim includes a patient identifier, date of visit, service code, diagnostic code, and the specialty of the physician providing the service. We identified all office visits made by cohort members to a PCP or psychiatrist within two years of the baseline cohort interview. Mental health-related visits to a PCP were identified using a validated algorithm based on the service and diagnosis codes found in the claims record. The positive predictive value for identifying a mental health related primary care visit using this algorithm is 84.9%; sensitivity and specificity are 80.7% and 97.0%, respectively[26]. Mental health-related visits to a psychiatrist were those claims submitted by a psychiatrist for a core mental health service[27].

In addition, for cohort members aged 65+ years at baseline, and thus covered by the Ontario Drug Benefit (ODB) Program, we ascertained use of prescription anti-depressants. A comprehensive list of all prescription medications used to treat depression was compiled with input from a clinical pharmacologist and psychiatrist (Additional File 1: Appendix 1). The ODB database was searched to identify prescriptions for anti-depressant medications in the two-year period following the baseline assessment. Whenever possible, prescriptions were linked to a physician database to determine the prescriber's specialty.

### Statistical Analysis

WOMAC and SF-36 scores were rescaled to a 0-100 scale. Individuals with probable depression (MH subscale score < 60) were compared to those without depression using chi-square tests (categorical characteristics), Wilcoxon rank sum tests (ordinal/skewed variables), and t-tests (normally distributed variables). The proportion with probable depression that experienced one or more mental-health related PCP visit was calculated with 95% confidence intervals.

For the full cohort, we examined the relationship between MH scores and mental health-related visits to a PCP or specialist using zero-inflated negative binomial (ZINB) regression to adjust for both over dispersion and the "excess" zeros in the data[28]. The ZINB model simultaneously models the contribution of the independent variables on: (1) the probability of having any mental health visits at all; and (2) the number of visits, given that the person has at least one visit. For those aged 65+ years at baseline, logistic regression was used to examine the probability that an individual received any mental health-related care (one or more mental-health related visits to a PCP or psychiatrist, or filling one or more prescriptions for an anti-depressant therapy).

Each model adjusted for other variables that have been shown to be related to health care use[13,29]: female sex, older age, urban versus rural region of residence, a greater number of comorbidities and worse general health status (SF-36 general health subscale score), lower income and education, residing in long term care, and greater OA severity. Education and income were included in the regression models as categorical variables. For each of these variables, people with missing information were retained in the analyses by including a separate 'missing' category. Adjusted models included interactions between the MH subscale score and other covariates, allowing the effect of mood to vary by subgroup. Analyses were conducted using SAS Version 9.2 (SAS Institute, Cary, North Carolina). A two-tailed level of significance of 0.05 was used.

## Results

### Baseline Characteristics

Participants with inflammatory arthritis (n = 186), missing MH scores (n = 63), or who died within the two years following the baseline interview (n = 159) were excluded; analyses are based on 2,005 (90.1%) cohort participants with OA. Participant characteristics are shown in Table 1: mean age was 70.7 years, and most were female (73.2%) and Caucasian (93.0%), with low income (52.4% reported an annual income ≤ $20,000) and low education (83.2% reported ≤ high school education). WOMAC pain, disability and summary scores indicated moderate-to-severe OA pain and disability. One-fifth (19.2%) reported 3 or more comorbid conditions.

**Table 1:** Baseline characteristics of the analysis cohort (n = 2,005)

Characteristic	Overall N = 2,005	Mental health score ≥ 60 N = 1,429	Mental health score < 60 N = 576	p-value *
**Sex (% female)**	73.2	72.2	75.5	0.13
**Age in years**: mean (S.D.)	70.7 (9.1)	71.2 (8.9)	69.7 (9.4)	0.002
**Region (% urban)**	43.5	41.5	48.4	0.005
**Income (%)**				< 0.0001
≤ $20,000	52.4	47.7	63.9	
> $20,000	30.2	33.2	22.6	
Missing	17.5	19.0	13.5	
**Education (%)**				< 0.0001
< high school graduation	35.7	33.0	42.4	
High school graduation	47.5	47.7	47.1	
Some post-secondary education	14.9	17.6	8.3	
Missing	2.0	1.8	2.3	
**Living arrangements (%)**				0.16
Lives alone	30.3	29.5	32.1	
Lives with others	66.4	67.5	63.7	
In long-term care	1.4	1.1	2.1	
missing	2.0	1.9	2.1	
**Race (%)**				0.082
Caucasian	93.0	93.1	92.7	
Non-Caucasian	3.7	3.2	4.9	
Missing	3.3	3.7	2.4	
**Number of comorbidities (%)**				< 0.0001
None	29.2	31.0	24.8	
1	30.6	32.4	26.0	
2	21.0	19.7	24.3	
3+	19.2	16.9	24.8	
**Body Mass Index**: mean (S.D.)	28.1 (5.4)	28.1 (5.2)	28.0 (5.8)	0.82
**SF-36 general health scale **/100: mean (S.D.)	49.2 (22.1)	54.7 (20.7)	35.5 (19.2)	< 0.0001
**WOMAC total score **/100: mean (S.D.)	40.3 (19.5)	37.5 (18.6)	47.3 (20.0)	< 0.0001
**WOMAC pain score **/100: mean (S.D.)	40.5 (21.7)	37.9 (21.0)	46.8 (21.9)	< 0.0001
**Mental Health Subscale score **/100: Mean (S.D.)	68.5 (20.4)	79.0 (11.3)	42.5 (13.2)	< 0.0001
**Self-reported depression**				
(% reporting ever depressed or other major mental illness)	16.4	7.2	39.2	< 0.0001
(% reporting treatment for depression or other major mental illness in past year)	9.2	3.2	24.1	< 0.0001

*P values comparing depressed to non-depressed. Fisher's Exact tests were used to compare binary characteristics, chi-square tests were used to compare characteristics with more than 2 categories, t-tests were used to compare normally distributed variables (WOMAC, SF-36, age).

### Prevalence and Correlates of "Depressed Mood"

Participants' mean MH score was 68.5 (SD 20.4); 576 (28.7%) had a score < 60, indicating probable depression. Among all participants, 329 (16.4%) self-reported 'ever' having been diagnosed or treated for depression or another major mental health condition, while 9.2% reported receiving treatment in the past year. Those classified as having probable depression were younger, more likely to reside in the urban region, reported lower income and less education, and had worse OA pain and disability and a greater number of comorbidities (all p < 0.05). Among the 576 participants with 'probable depression', 226 (39.2%) reported 'ever' having been diagnosed or treated for a mental health problem (24.1% in the past year) compared with 7.2% (3.2%), respectively, among those without probable depression (both p < 0.0001; see Table 1).

### Mental Health-Related Physician Visits Over Two Years

Most study participants (95.2%) experienced one or more PCP visit over the two-year study period; in total, cohort members experienced 34,000 PCP visits. Over one-quarter (28.9%) experienced one or more mental health-related PCP visit (Table 2). Fewer participants (5.3%) experienced one or more visit to a psychiatrist (n = 106). Among those with probable depression, 39.1% experienced a mental health-related PCP visit and 10.1% saw a psychiatrist. Overall, 618 participants (30.8%) experienced one or more mental health related physician visit (PCP or psychiatrist) in the two-year period (42.5% of those with depressed mood). In those who experienced a mental-health related physician visit, only 19 (5.1%) of the 373 with depressed mood saw *only *a psychiatrist.

**Table 2:** Primary care visits and mental health care received over two years in those with and without depressed mood

Visits - Full Sample	Overall N = 2,005	Mental health score ≥ 60 N = 1,429	Mental health score < 60 N = 576	p-value*
Visits to a primary care physician				
% (CI^†^) with at least one visit	95.2 (94.2 - 96.1)	94.5 (93.4 - 95.7)	96.7 (95.2 - 98.2)	0.05
Total number of visits to a primary care physician in the first 2 years: median (inter-quartile range)	13 (7-23)	13 (7-21)	16 (9-26)	< 0.0001
Mental health visits to a primary care physician				
% (CI^†^)with at least one mental health visit	28.9 (26.9 - 30.9)	24.8 (22.5 - 27.0)	39.1 (35.1 - 43.1)	< 0.0001
Number of visits, for those who had at least one visit:				
median (inter-quartile range)	2 (1-3)	1 (1-3)	2 (1-4)	0.0007
Visits to a psychiatrist				
% (CI^†^) with at least one visit	5.3 (4.3 - 6.3)	3.4 (2.4 - 4.3)	10.1 (7.6 - 12.5)	< 0.0001
Number of visits, for those who had at least one visit:				
median (inter-quartile range)	3 (1-13)	3 (1-10)	5 (2-20)	0.11
Visits to a PCP *and/or *psychiatrist				
% (CI^†^) with at least one visit	30.8 (28.8 - 32.8)	26.1 (23.8 - 28.4)	42.5 (38.5 - 46.6)	< 0.0001
Number of visits, for those who had at least one visit:	2 (1-4)	2 (1-3)	2 (1-6)	< 0.0001
median (inter-quartile range)				
				

**Visits - Those aged 65+ years at baseline**	**Overall** **N = 1,425**	**Mental health score ≥ 60** **N = 1,049**	**Mental health score < 60** **N = 376**	p-value*

Visits to a primary care physician				
% (CI^†^) with at least one visit	95.1 (94.0 - 96.2)	94.3 (92.9 - 95.7)	97.3 (95.7 - 99.0)	0.018
Total number of visits to a primary care physician in the first 2 years: median (inter-quartile range)	14 (8-23)	13 (7-22)	17 (9-27)	< 0.0001
Mental health visits to a primary care physician				
% (CI^†^)with at least one mental health visit	28.1 (25.7 - 30.4)	24.9 (22.3 - 27.5)	37.0 (32.1 - 41.9)	< 0.0001
Number of visits, for those who had at least one visit:	2 (1-3)	1 (1-3)	2 (1-4)	0.0087
median (inter-quartile range)				
Visits to a psychiatrist				
% (CI^†^) with at least one visit	4.6 (3.5 - 5.7)	3.2 (2.1 - 4.2)	8.8 (5.9 - 11.6)	< 0.0001
Number of visits, for those who had at least one visit:	3 (1-10)	3 (1-9)	4 (1-15)	0.23
median (inter-quartile range)				
Any mental health care visit (to a PCP or psychiatrist)				
% (CI^†^) with at least one visit	30.0 (27.6 - 32.3)	26.1 (23.5 - 28.8)	40.7 (35.7 - 45.7)	< 0.0001
Number of visits, for those who had at least one visit:	2 (1-4)	2 (1-3)	2 (1-6)	0.0024
median (inter-quartile range)				
Prescriptions for antidepressants				
% (CI^†^) who filled at least one prescription	23.1 (20.9 - 25.3)	18.4 (16.1 - 20.7)	36.2 (31.3 - 41.0)	< 0.0001
Number of prescriptions filled, for those who filled at	6 (2-11)	6 (2-10)	7 (2-12)	0.064
least one: median (inter-quartile range)				
Any mental health care				
% (CI^†^) with at least one mental health visit to a PCP or at least one visit to a psychiatrist or filling at least one prescription for an antidepressant	40.6 (38.1 - 43.2)	34.9 (32.0 - 37.8)	56.7 (51.6 - 61.7)	< 0.0001

*P values comparing depressed and non-depressed people. Wilcoxon rank sum tests were used to compare the numbers of visits; a Fisher's Exact test was used to compare the percentage of people having at least one mental health visit.

^†^CI = 95% confidence interval

### Mental Health Care Use (Physician Visits and Prescriptions for Anti-Depressants) in Those 65+ Years at Baseline

Of the 2005 study participants, 1425 (71.1%) were aged 65+ years at baseline and thus eligible for drug benefits coverage; of these, 376 (26.4%) met the criteria for probable depression. Mental-health related physician visits and prescriptions for anti-depressants are shown in Table 2. Overall, 329 participants (23.1%) filled one or more prescriptions for an anti-depressant; in total, 2540 prescriptions were filled. Specialty was missing for 14.7% of these prescriptions; where not missing, 86.4% of the prescriptions were written by a PCP, 8.2% by a psychiatrist, and 2.8% by a geriatrician or general internist. Individuals with probable depression were more likely to fill a prescription (36.2% versus 18.4%, p < 0.0001). Among those 65 years and older at baseline, 579/1,425 (40.6%) received *any *mental health care (saw a PCP or psychiatrist and/or filled a prescription for an anti-depressant); 56.7% with probable depression received care.

### Predictors of Mental Health-Related Physician Visits (Full Sample)

Unadjusted for other factors, a 10-point worsening of the MH score was associated with increased odds of having one or more mental health-related physician visit (odds ratio, OR, 2.14, p = 0.03) (Table 3). In the adjusted model, the likelihood of experiencing one or more mental health-related physician visit was significantly and independently associated with female sex (adjusted OR women vs. men = 5.87, p = 0.005) and MH score (adjusted OR per 10-point decrease in MH score = 1.63, p = 0.003). Among those who experienced at least one mental health visit, significant, independent predictors of the *number *of mental health visits were MH score, region of residence, and level of education. Every 10-point deterioration in MH score was associated with a 22.4% increase in the number of mental-health visits (p < 0.0001). The number of mental health-related visits was 106% higher among urban than rural residents (p < 0.0001), and 58.0% higher among those with some post-secondary education than among those who had not completed high school.

**Table 3:** Predictors of receiving one or more mental health related physician visit (PCP or Psychiatrist), and of the total number of visits made during the 2-year period

Model 1: Regression Model with SF-36 Mental Health Score as the Only Independent Variable			
**Odds of having at least one mental health visit**	**odds ratio**	**95% confidence interval**	**p-value**
SF-36 mental health per 10-point deterioration	2.14	1.08 to 4.26	0.031
**Predictors of number of visits, given that one has visits**	**Change in number of mental health visits**	**95% confidence interval**	**p-value**
SF-36 mental health per 10-point deterioration	25.3%	17.6% to 33.4%	< 0.0001

Model 2: Regression Model for the Effect of SF-36 Mental Health Score, Adjusted for Additional Covariates*			

**Odds of having at least one mental health visit**	**odds ratio**	**95% confidence interval**	**p-value**
SF-36 mental health score per 10-point deterioration	1.63	1.18 to 2.24	0.0027
Female sex (baseline is male)	5.87	1.73 to 20.0	0.0046
**Predictors of number of visits, given that one has any visits)**	**Change in number of mental health visits**	**95% confidence interval**	**p-value**
SF-36 mental health per 10-point deterioration	22.4%	15.1% to 30.2%	< 0.0001
Urban region (reference is rural)	106%	65.7% to 157%	< 0.0001
Education (reference is < high school graduation)			0.046
High school graduation	13.5%	-10.9% to 44.6%	0.31
Some post-secondary education	58.0%	12.5% to 122%	0.0082
Missing	40.6%	-36.8% to 213%	0.40

* Additional covariates that were considered in the regression analysis were: age, sex, number of comorbid conditions, SF-36 general health score, WOMAC total score and pain subscale, education, income, living arrangements, marital status, region, and race. An interaction between age and sex was also included. Interactions between the mental health score and the other variables were included in order to allow the effect of mental health to vary by sub-group. All significant covariates are reported.

### Predictors of Receiving Any Mental Health Care (Physician Visit or Anti-Depressant Prescription) *(Those 65+ Years at Baseline)*

**Figure 1:**
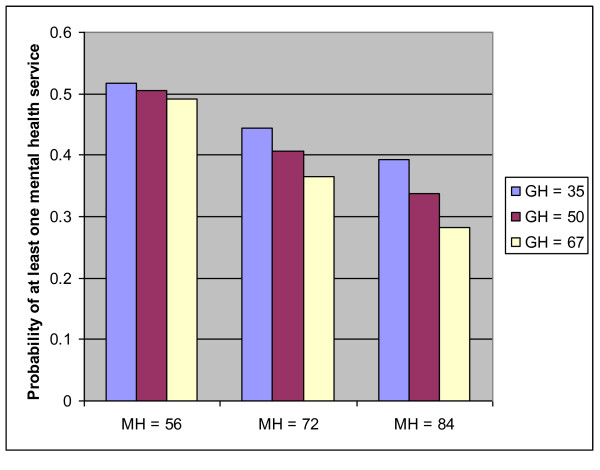
**Probability of receiving at least one mental health service for a woman aged 75 years**. This figure illustrates the effect of the significant interaction between mental health score and general health score on the predicted probability of receiving at least one mental health service (visit to a PCP or psychiatrist, or at least one prescription for an antidepressant). The figure shows the predicted probabilities for a women aged 75 years (the average age for those who were over the age of 65), living in the rural area, for representative values of the mental health and general health scores (the values chosen are the 25^th ^percentile, median, and 75^th ^percentile for each score). The probabilities are lower for men, higher for those in the urban area, and higher for those younger than 75 years (and lower for those older than 75 years). The chart shows that, holding GH score constant, the probability of at least one mental health service increases with deteriorating MH score (lower MH scores indicate worse mental health). Holding MH score constant, the probability of at least one service increases with deteriorating general health (for GH, higher scores indicate better self-reported health status). The effect of worsening general health status is non-significant in the setting of a poor MH score.

Unadjusted for other factors, among those 65 years or older at baseline, a 10-point worsening of the MH score was associated with increased odds of receiving one or more mental health service (OR 1.30, p < 0.0001) (Table 4). In the adjusted model, significant, independent predictors of the likelihood of experiencing one or more mental health service were: younger age (adjusted OR per 10-year increase in age = 0.80, p = 0.01), female sex (adjusted OR women vs. men = 1.79, p < 0.0001), region of residence (adjusted OR urban vs. rural = 1.36, p = 0.008), and an interaction between MH score and self-reported general health status (p-value for the interaction = 0.0009), such that the likelihood of receiving at least one mental health service was greatest for those with low self-rated general health *and *worse MH scores, but the effect of worsening MH scores declined with declining general health status (Figure 1).

**Table 4:** Logistic regression model for the probability of at least one mental health service for those over the age of 65

Model 1: Regression Model with SF-36 Mental Health Score as the Only Independent Variable (R-square = 0.080)			
**Independent variable**	**Odds Ratio**	**95% confidence interval**	**p-value**
SF-36 Mental Health score per 10-point deterioration	1.3	1.23 to 1.38	< 0.0001

Model 2: Regression Model for the Effect of SF-36 Mental Health Score, Adjusted for Additional Covariates* (R-square = 0.124)			

**Independent variable**	**Odds ratio**	**95% confidence interval**	**p-value**
Age per 10-year increase in age	0.8	0.68 to 0.95	0.012
Female sex (baseline is male)	1.79	1.37 to 2.34	< 0.0001
Urban region (reference is rural)	1.36	1.08 to 1.71	0.0083
Interaction between mental and general health†			0.0009
Effect of a 10-point deterioration in general health score	1.04	0.97 to 1.11	0.32
when mental health score = 56 (25^th ^percentile for mental health score; poor mental health)			
Effect of a 10-point deterioration in general health score when mental	1.11	1.05 to 1.18	0.0007
health score = 72 (median mental health score)			
Effect of a 10-point deterioration in general health score when mental	1.15	1.10 to 1.21	< 0.0001
health score = 84 (75^th ^percentile mental health score; good mental health)			
Effect of a 10-point deterioration in mental health score when general	1.2	1.12 to 1.29	< 0.0001
health score = 35 (25^th ^percentile general health score; poor health)			
Effect of a 10-point deterioration in mental health score when general	1.28	1.20 to 1.37	< 0.0001
health score = 50 (median general health score)			
Effect of a 10-point deterioration in mental health score when general health score = 67 (75^th ^percentile general health score; good health)	1.38	1.26 to 1.52	< 0.0001

* Additional covariates that were considered in the regression analysis were: age, sex, number of comorbid conditions, SF-36 general health score, WOMAC total score and pain subscale, education, income, living arrangements, marital status, region, and race. An interaction between age and sex was also included. Interactions between the mental health score and the other variables were included in order to allowed the effect of mental health to vary by sub-group. All significant covariates are reported.

† The significant interaction between the SF-36 mental health score and the SF-36 general health score means that both scores are significant predictors of the number of mental health visits, and that the effect of the mental health score varies with general health and the effect of the general health score varies with mental health. To illustrate the form of the interaction, the effect of increasing mental health score is presented for each of 3 representative ages (the 25th percentile age, the median age, and the 75^th ^percentile age); and the effect of increasing age is presented for each of 3 representative mental health scores (the 25^th ^percentile score, the median score, and the 75^th ^percentile score). For younger patients, the odds of a mental health visit decreases as the score improves; whereas for older patients, the odds of a mental health visit are not affected by the score. For patients with the worst (lowest) mental health scores, the odds of a mental health visit decrease with increasing age, whereas for patients with better (higher) mental health scores, the odds are less affected by age.

## Discussion

In a population cohort with symptomatic hip and knee OA, we examined the relationship between depressed mood, evaluated using the SF-36 MH score, and mental health-related health care use. Controlling for potential confounders, worsening MH scores were significantly and independently predictive of a greater likelihood of receiving mental health services. However, consistent with previous studies in other clinical populations[17,30], and despite mounting evidence of a strong association between chronic pain conditions, like arthritis, and depression[8,9,31,32], substantial care gaps remained. Fewer than half with depressed mood, as we defined it, experienced one or more mental health-related physician visit to a PCP or psychiatrist; among those aged 65+ years, who were eligible for drug benefits coverage, the proportion receiving any care (physician visit and/or prescription for an anti-depressant) was only modestly higher at 56.7%.

Among our participants, more than one-quarter (29%) had MH scores below our cut-point, indicating probable depression. Probable depression was more common among those who were younger, resided in the urban region, had lower income and education, greater OA severity and greater comorbidity. These findings are consistent with those of others. A cross-sectional analysis of the 2002 US National Health Interview Survey[33] found that 26.2% with physician-diagnosed arthritis reported frequent anxiety or depression in the previous 12 months; 5.6% met criteria for 'serious psychological distress', which was significantly and independently associated with younger age, lower socioeconomic status, divorce/separated marital status, greater pain and functional limitations, and comorbidity. A smaller UK study found that 40.7% of 54 participants with lower limb OA[34] met criteria for clinically significant anxiety or depression, with worse scores significantly related to greater OA pain.

Depressed mood in the setting of chronic pain has been linked with greater pain intensity, anxiety[35], sleep disturbances, decreased energy, decline in cognitive function and poor medication adherence[36], each of which may increase health care use. In the current study, depressed mood predicted a greater number of visits to both PCPs and psychiatrists and a greater likelihood of receiving an anti-depressant prescription. Katon et al. [37] similarly found that, among primary care patients aged 60+ years, and controlling for age, sex, and comorbidity, inpatient and outpatient health care utilization, including PCP and specialty medical visits and prescriptions for anti-depressants, were higher among those who did versus did not screen positive for clinical depression on a structured clinical interview. However, consistent with our findings, only 45% of the individuals with depression experienced any mental health care.

Although women were not more likely than men to be classified as having probable depression, women were more likely to receive mental health care. A similar relationship has been shown by others[19,29] and may be related to a greater propensity to seek treatment for mental health problems among women than men[38].

Among those 65 and older at baseline, the probability of receiving mental health care *decreased *with increasing age. One potential explanation is that the greater comorbidity that accompanies increasing age is perceived as precluding the safe use of anti-depressant therapies. However, among our study participants, while the number of reported comorbid conditions did increase with increasing age, age was a significant predictor of the probability of receiving mental health care and remained significant even after controlling for the number of comorbid conditions, suggesting that the effect of age was not simply as a proxy for greater comorbidity. Other potential explanations include under-recognition of depression among older adults, possibly resulting from differences in the clinical presentation of depression by age, and/or a higher threshold for seeking mental health care among older individuals[39,40]. Further, self-reported general health status modified the relationship between MH scores and likelihood of receiving mental health care. Among those with relatively good general health status, worsening mental health was associated with an increased likelihood of receiving mental health care, but as general health status declined, this effect was attenuated. One explanation for this finding is that, in the setting of multiple medical conditions, for which poor self-reported general health status may be a proxy, the management of some conditions may be neglected if others consume attention[41]. Alternatively, these individuals may have their mental health care needs addressed within the context of physician visits coded for their other health care problems. Further research is warranted to disentangle the influences of general health and mental health status on provision of mental health care.

Among those who received at least one mental health-related physician visit, the number of visits experienced was significantly greater in urban residents and those with more education. It has previously been shown that urban residence is associated with greater use of mental health specialist services[42], likely related to greater access to these services. The association with higher socioeconomic status is concerning in light of the documented higher risk for depression among older adults with lower socioeconomic status[33]. This finding may reflect differences by socioeconomic status in perceptions of need, health-seeking behaviours, likelihood of receiving treatment from a physician, and adherence to recommended therapies once prescribed. Additional research is warranted to determine if inequities in care provision exist.

Taken together, our findings suggest under-treatment of depressed mood among older adults with painful OA. Identified barriers to the diagnosis and treatment of depression in the primary care setting, where most mental health care was received by our participants, include: barriers to help-seeking for mental health issues due to the stigma attached to these conditions[38,43] and the perception that a depressive state is a normal part of aging[44]; physicians' attitudes, knowledge and skills with respect to mental health diagnosis and management[17,45]; the complexity of depression management in the elderly[17,45,46]; and difficulty discriminating the clinical symptoms of OA from those of depression[39,40]. Strategies are needed to address these barriers as effective therapies exist [47,48] since, in the setting of painful OA, improved treatment of depression may reduce not only depressive symptoms, but also arthritis pain, activity limitations, and overall quality of life[16].

This was a retrospective cohort study in which we utilized previously completed questionnaires, which incorporated the SF-36. As such, we did not have access to the medical records of the participants, nor would we be able to retrospectively evaluate whether or not the participants we categorized as having 'probable depression' met DSM-IV criteria for clinical depression at that time. For this reason, we have been careful to use the term 'depressed mood' as opposed to 'clinical depression' to describe these individuals. However, despite this limitation, we would argue that individuals who have sufficient symptoms of depression to meet our criteria for 'probable depression' would warrant a closer look by the family doctor and/or a referral to a specialist, even if a psychiatrist decided that the patient did not meet the DSM-IV definition. Study strengths include the large sample recruited from the community and use of linked survey and administrative data. However, there are also potential study limitations. First, we defined depressed mood using a validated cut-point on the SF-36 MH subscale, shown to have 78.7% sensitivity and 72.1% specificity for clinical depression based on clinical interview using the MINI-MDE module[49]. Thus, there remains the potential for misclassification of depressed mood in our cohort. Second, the validated algorithm used to identify mental health-related PCP visits using administrative data has high specificity, but only 80% sensitivity [26]. Thus, we may have underestimated mental health-related PCP visits, and thus overestimated the depression-care gap. Third, since Ontario drug benefits are restricted to individuals aged 65 years and older, we were only able to examine use of medications for depression among those aged 65+ years at baseline. However, this subgroup represented over 70% of our total sample. Fourth, for almost one-third of anti-depressant prescriptions identified in this cohort subgroup (30.4%), the 'days supplied' variable was missing; thus, we relied on the filling of a prescription as a proxy for the participant taking the medication. Finally, we made the assumption that anti-depressants were prescribed for the management of depressed mood; some may have been prescribed instead for the management of chronic arthritis pain and/or associated fibromyalgia. Both these decisions may have resulted in over-estimation of receipt of mental health care.

## Conclusions

Among older adults living with painful OA, depressed mood is common and associated with increased mental health-related health care, including visits to primary care physicians and psychiatrists, and prescriptions for anti-depressant therapies. Despite this, as many as half with comorbid depressed mood received no mental health care over the two year study period, indicating under-diagnosis and under-treatment. Our results further suggest that the care gap may be relatively greater among men, those living in rural regions, those with less education, and the very old. As effective therapies exist for the treatment of depression among older adults[47,48] and effective treatment of depression in OA may reduce pain and improve quality of life[19], the documented care gap is concerning. Our findings underscore the need for improved identification and management of depressed mood in the growing population with painful OA.

## Competing interests

The authors declare that they have no competing interests.

## Authors' contributions

Study design and concept: YG, RC, JH, GAH. Acquisition of subjects and data: YG, RC, JH, GAH. Analysis and interpretation of data: YG, RC, JH, GAH. Preparation of manuscript: YG, RC, JH, GAH. All authors read and approved the final manuscript.

## Pre-publication history

The pre-publication history for this paper can be accessed here:

http://www.biomedcentral.com/1471-244X/11/147/prepub

## Supplementary Material

Additional file 1 **Appendix 1**. List of Prescription Medications Considered Treatment for Depression. Click here for file
