# Supplementary material for: A prospective study of mental health care for comorbid depressed mood in older adults with painful osteoarthritis
Source: BMC Psychiatry. 2011 Sep 12;11:147. doi: 10.1186/1471-244X-11-147 (PMC3184052; doi:10.1186/1471-244X-11-147)
Supplement: Additional file 1 — Appendix 1. List of Prescription Medications Considered Treatment for Depression. [file 1471-244X-11-147-S1.DOC]

**Appendix 1. List of Prescription Medications Considered Treatment for Depression***

| **Drug Class** | **Generic Drug Name** |
| --- | --- |
| Irreversible MAO | Phenelzine Sulfate |
|  | Tranylcypromine Sulfate |
|  |  |
| Reversible MAO | Moclobemide |
|  |  |
| Selective Serotonin Reuptake Inhibitor | Citalopram Hydrobromide |
|  | Escitalopram oxalate |
|  | Fluoxetine HCl |
|  | Fluvoxamine maleate |
|  | Paroxetine HCl |
|  | Sertraline HCl |
|  |  |
| Serotonin-Norepinephrine Reuptake Inhibitor | Venlafaxine HCl |
|  |  |
| Tricyclic Antidepressant | Amitriptyline HCl |
|  | Amitroptyline pamoate |
|  | Amoxapine |
|  | Clomipramine HCl |
|  | Desipramine HCl |
|  | Doxepin HCl |
|  | Imipramine |
|  | Nortriptyline HCl |
|  | Protriptyline HCl |
|  | Trimipramine |
|  |  |
| Other | Bupropion HCl |
|  | Maprotiline HCl |
|  | Mirtazapine |
|  | Trazodone HCl  100mg and 150mg strengths only |

* Medications listed are those contained in the Ontario Drug Benefit Plan formulary.
